# A De Novo Pharmacist-Family Physician Collaboration Model in a Family Medicine Clinic in Alberta, Canada

**DOI:** 10.3390/pharmacy9020107

**Published:** 2021-05-28

**Authors:** Hoan Linh Banh, Andrew J. Cave

**Affiliations:** Department of Family Medicine, Faculty of Medicine and Dentistry, University of Alberta, 6-10 University Terrace, Edmonton, AB T6G 2C8, Canada; andrew.cave@ualberta.ca

**Keywords:** clinical pharmacy, clinical pharmacist, family medicine, family physician

## Abstract

Collaborative practice in health-care has proven to be an effective and efficient method for the management of chronic diseases. This study describes a de novo collaborative practice between a pharmacist and a family physician. The primary objective of the study is to describe the collaboration model between a pharmacist and family physician. The secondary objective is to describe the pharmacist workload. A list of patients who had at least one interaction with the pharmacist was generated and printed from the electronic medical record. There were 389 patients on the patient panel. The pharmacist had at least one encounter with 159 patients. There were 83 females. The most common medical condition seen by the pharmacist was hypertension. A total of 583 patient consultations were made by the pharmacist and 219 of those were independent visits. The pharmacist wrote 1361 prescriptions. The expanded scope of practice for pharmacists in Alberta includes additional prescribing authority. The pharmacists’ education and clinical experience gained trust from the family physician. These, coupled with the family physician’s previous positive experience working with pharmacists made the collaboration achievable.

## 1. Introduction

The World Health Organization (WHO) defines collaborative practice in health-care as “multiple health workers from different professional backgrounds providing comprehensive services by working with patients, their families, carers and communities to deliver the highest quality of care across settings” [1]. This practice has proven to be effective and efficient for the management of chronic diseases [2]. It has been shown to benefit patients, health professionals and health organizations. Many regulatory agencies in Medicine and Pharmacy in Canada deemed collaborative practice essential for patient care [3,4,5,6]. Evidence has shown that pharmacist-led services, such as health screenings, smoking cessation management, vaccine administration and comprehensive medication review, can improve patient outcomes and reduce medical costs [7,8,9,10]. Pharmacists in Canada have the training and competency to provide direct patient care through collaboration with physicians. Pharmacists across Canada are expanding their roles to optimize patient care, [11] and have the training and competence to provide direct patient care through collaboration with physicians. The expanded scope of pharmacy practice in the province of Alberta, Canada is unique. The 2012 Pharmacy Act approved pharmacists in Alberta to prescribe all Schedule 1 drugs independently [12]. Studies have shown the positive reception of physicians toward collaboration with pharmacists [13,14,15]. Community pharmacists have demonstrated that, in collaboration with family physicians, they can enhance medication adherence and comprehensive medication assessment [14,16]. Numerous studies have demonstrated pharmacist–physician collaboration in disease management, such as hypertension, anti-coagulation and diabetes, in a primary care setting results in improved patient outcomes [17,18,19,20,21,22,23,24,25,26,27,28,29,30,31,32]. Family medicine focuses on the individual as part of the family and community. Family physicians provide care across the entire spectrum of care independent of age, gender or morbidity [33]. They establish a close, long-term physician/patient relationship throughout the patient’s life. The care is provided from birth to death. Family physicians deliver care in various settings, including private offices, hospital, long-term care facilities and the patient’s home. In addition, family physicians play a crucial role in health promotion and illness prevention, coordinating care with other specialties and health professionals. They also advocate on behalf of their patients’ care and services in all parts of the health care system. For example, they advocated for no smoking in restaurants or for making wearing seat belts mandatory in Canada. Family physician roles are different from those of other primary care physicians such as primary care pediatricians, internists or psychiatrists who have a limited range of practice (Table 1).

To the best of our knowledge, there has been no documented collaboration between a pharmacist and family physicians in providing direct patient acute care in a primary care setting. This study describes a de novo collaborative practice between a pharmacist and a family physician in a family medicine clinic. The primary objective of the study is to describe the collaboration model between a pharmacist and family physician in a family medicine clinic. The secondary objectives are to describe the workload for the pharmacist.

## 2. Materials and Methods

At the time of the study, The Kaye Edmonton Family Medicine Clinic is part of the Edmonton West Primary Care Network. The clinic has 11 family physicians (9 FTE), 3 × 0.3 FTE nurse, 0.8 FTE chronic disease management nurse, a 0.1 FTE dietician, a 0.1 FTE social worker, 0.1 FTE respiratory therapist and a 0.4 FTE pharmacist. Three of the family physicians and the pharmacist are Faculty Members of the University of Alberta, Department of Family Medicine. The rest of the family physicians are private practitioners. The family physician Faculty Members are compensated by Alberta Health through an Alternate Relationship Plan, a negotiated contract. The pharmacist’s salary is paid by the University of Alberta. Both the pharmacist and the participating family physician allocated four half days a week (40%) of their time in the clinic. The family physician had an accompanying family medicine resident most clinic days. Prior to the collaboration, both the family physician and pharmacist agreed to a list of criteria for the collaboration. The pharmacist would not see patients under 18, conduct physical exams, perform short procedures or other procedures such as Papanicolaou (PAP) test. The pharmacist would independently see patients who required prescription refills, comprehensive medication reviews, or follow up visits for stable chronic conditions such as hypertension, diabetes, mental health, chronic pain, dyslipidemia and hypothyroidism. Each patient visit is scheduled in the family physician schedule in 20 min time slot except for physical exams or short procedures, which are allocated more time. At the start of the clinic, both the family physician and pharmacist review the patient list and decide which patient will be seen by the pharmacist independently as above. This will expedite the clinic flow as the pharmacist would be able to discharge the patient after the visit without a consultation with the family physician. As for the patients who would be seen collaboratively, there is a brief discussion of the approach to provide care to the patient based on the reason for the visit. The pharmacist would see the patient and gather pertinent information and assess and develop a plan. The plan is discussed with the family physician. Both the family physician and the pharmacist would deliver the plan to the patient. If the patient required a follow up appointment, the pharmacist would be able to see that patient independently.

A list of patients of the family physician was generated and printed from the electronic medical records on 8 June 2020. The pharmacist examined the records of the listed patients for the documentation of encounters with the pharmacist between April 2014 and March 2020, using Connectcare^®^, eClinician^®^ and Netcare^®^/PIN, which are the electronic medical records used by the clinic successively over that period. All patients with at least one encounter with the pharmacist were included in the study. An encounter is defined as a clinic visit or a prescription written. Clinic visits are divided into independent or shared visits. An independent visit is defined as the pharmacist seeing the patient without a patient consultation with the family physician, and a shared visit is defined as a visit where the pharmacist saw the patient together or in consultation with the family physician. Both Connectcare^®^ and the preceding eClinician^®^ databases were used to extract visit information. On 8 November 2019, the clinic launched Connectcare^®^ to replace eClinician^®^.

Each progress note from every patient was reviewed. If the progress note included, “patient seen with Dr. X” and/or there is an addendum from the family physician, it is considered a shared visit. Otherwise, the visits are considered independent visits. The number of prescriptions were extracted from the Pharmaceutical Information Network (PIN), which is a comprehensive provincial database that contains all prescriptions actually dispensed for a patient in Alberta. It includes the medication name, dose, frequency, quantity dispensed, date dispensed and prescriber’s name. The database is uploaded by the community pharmacies daily. The study received approval from The University of Alberta Research Ethics Board on 1 June 2020 (Pro00101144).

## 3. Results

The top four medical conditions for which the pharmacists saw the patients were HTN, hypothyroidism, diabetes mellitus and chronic pain. On 8 June 2020, the family physician had 389 patients who listed him as their general practitioner. A total of 90 patients were inactive—which means they have not seen the family doctor for at least 5 years—37 patients were under 18 years old and 11 patients were between 40 and 65, who only came to the clinic for physical exams. Between April 2014 and March 2020, the pharmacist had at least one encounter with 159 out of 251 eligible patients. There were 83 (53.5%) females and the average age of the patients was 62 years. The most common medical condition seen by the pharmacist was hypertension (HTN). The patient characteristics are summarized in Table 2. Other conditions that the pharmacist saw the patients for are gout, gastroesophageal reflux disease and insomnia.

**Figure 1:**
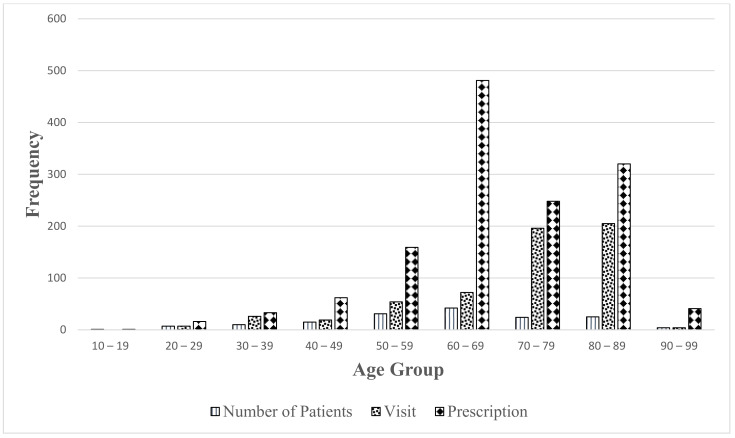
Graph represents number of visits and prescriptions by age group.

A total of 583 patient consultations were made by the pharmacist and 219 (38%) of those visits were seen independently. The average number of visits made is four per patient and the range is between zero and 47 visits per patient over six years. The average number of independent visits is 1.5 and the range is between zero and eight visits per patient over six years. The pharmacist wrote 1361 prescriptions for 159 patients over six years. The average number of prescriptions written was nine and the range was from zero to 65 prescriptions per patient. Figure 1 summarizes the number of patients, number of visits, and number of prescriptions for each age group over six years. Table 3 is a summary of the patient visits and prescriptions written by the pharmacist. The pharmacist interventions included:

1. Monitored blood pressure and adjusted antihypertensives as appropriate;
2. Tapered or titrated antidepressants or requested a referral to mental health counselling;
3. Monitored thyroid function tests and adjusted medications as appropriate;
4. Recommended or prescribed appropriate antibiotics for community infections such as otitis media, urinary tract infection or cellulitis;
5. Adjusted dosage or discontinued medication due to organ dysfunction or failure such as renal insufficiency;
6. Tapered or titrated opiates;
7. Tapered benzodiazepines;
8. Deprescribed proton pump inhibitors;
9. Adjusted dosage of medications for diabetes.

## 4. Discussion

Because the expanded scope of practice for pharmacists in Alberta, Canada includes additional prescribing authority, this allowed the pharmacist the opportunity to manage these medical conditions independently. In 2014, 41% of the visits seen by the pharmacist were independent and it began to trend downward for four years. The reason is that, in 2014, most of the visits were a comprehensive medication review for which the pharmacist could see the patients independently. As the pharmacist saw more patients in the subsequent years, the patients were more complex and required consultations with the family physicians. As seen in 2019, the number of independent visits began to increase because most of the visits were with patients with stable chronic conditions returning for follow up visits. There is a decreased number of total visits in 2016 and 2017. This is because the pharmacist was teaching and training pharmacists in China and, as a result, the pharmacist had less clinic time in those two years. The number of total visits increased starting in 2018. The number of prescriptions written by the pharmacist increased year by year. In 2019, the life expectancy in Canada was 81 years old. As indicated in Figure 1, the distributions are not normal in character. Visits and prescriptions increase with age up to age 90 with the exception of the excessive number of prescriptions in the 60–69 age group. The majority of the patients are between 60 and 89 years old which is a good representation of the general population in Canada. Patients in this age group have multiple co-morbidities and they require more office visits and prescriptions.

Several studies have described the role of pharmacists in primary care settings [35,36]. The pharmacists in these studies provided direct patient care as consultants for medication-related issues. This means that the patients were referred to the pharmacists to provide specific care identified by the family physicians unlike in this study.

In addition, the reimbursement of the pharmacists in the primary care settings is paid by the government, which is not the case for family physicians who practice in most private settings. The uniqueness of the collaboration model described in this study is that both the pharmacist and family physician have a pre-determined set of criteria for the patients that the pharmacist would provide care for, either independently or in collaboration during the same clinic visit. Moreover, pharmacists in Alberta, Canada could prescribe medications that are not narcotics or controlled substances. This allows the pharmacist to initiate or discontinue a medication without the approval of a physicians as long as the care is documented and communicated with the physician.

The partnership described in this review was possible for many reasons. First, the family physician is not reimbursed by a fee for service and the pharmacist was paid by the University of Alberta. Often, the major barrier to forming a collaborative practice between the family physician and a pharmacist is the lack of a reimbursement model [37]. In addition, family physicians are not aware and not confident of the education and training that pharmacists have received, which leads to hesitation in forming collaborative practice [38]. The pharmacist in this study received a Doctor of Pharmacy degree and a Primary Care Specialty residency post-doctorate. Furthermore, the pharmacist had over 25 years of practice experience in diverse settings such as the intensive care, hospital pharmacy, community pharmacy and a poison control centre. Most importantly, the family physician had both experience of, and a very good professional relationship with, the health care team and community pharmacists while practicing in the UK. As a result, he is very receptive to developing a multidisciplinary practice in the clinic. In addition, the clinic itself has a structure that allows for multidisciplinary care.

### 4.1. Strength

To the best of our knowledge, from the literature, this is the first study that describes a de novo collaborative practice between a family physician and pharmacist in family medicine. Other studies have looked at the role of the clinical pharmacist in the hospital setting, particularly in the ICU [39,40,41] and in the avoidance of adverse drug reactions and medication errors in primary care settings [42,43,44]. There have been several studies in primary care examining the role of the pharmacist in the management of individual conditions (such as anticoagulation, dyslipidemia or hypertension, managing asthma or diabetes) [45,46,47]. We have described here the development of a permanent pharmacist/physician collaboration providing shared direct patient care in a family medicine setting. The two work together at the same time rather than on separate lists of patients. Family medicine prides itself on the comprehensiveness of the care provided to patients but this model adds to the knowledge and skills that go beyond those which either professional can deliver alone.

This model also provides pharmacy learners with the opportunity to interact with a family physician and family medicine residents. The experience could be extended to community or primary care pharmacy practice. In addition, the family medicine residents are exposed to a working environment with a pharmacist in the clinic and this may encourage future family physicians to collaborate with pharmacists to provide a comprehensive, expanded primary care.

### 4.2. Limitation

There are several limitations in the study. This is a retrospective patient chart review conducted by the pharmacist. The list of patients generated included only active patients as of 8 June 2020. Any patients who passed away, or transferred or moved out of the province prior to 8 June 2020 were not accounted for. When the clinic switched to a new software, Connectcare^®^, not all records were transferred from the old database eClinician^®^. As a result, some visit records could be missed. Not all prescriptions in the PIN database had a prescriber’s name. Some pharmacies assigned “prescribers unknown” when they did not have the pharmacist listed as a prescriber in their system. This results in an unknown number of prescriptions not accounted for. In the case where the prescriber was assigned as “prescriber unknown”, those prescriptions were included in the study. Lastly, it was not possible to distinguish new prescriptions initiated by the pharmacist from refill prescriptions on the PIN database.

We have only described here the structure and process involved and, as yet, we have not had access to health outcome measures, such as reduction of morbidity and mortality, patient satisfaction or effects on learners, but these are our plans for future studies.

There are certainly questions surrounding the generalizability of this model outside the stably funded academic teaching practice and the high level of experience and training of the pharmacist, but there are elements that are transportable. Alberta is the only province with such an advanced expanded scope of practice for pharmacists, but this is changing across the country. Some primary care physicians would like to have a pharmacist work FOR them, filling all the repeat prescriptions and completing comprehensive medication reviews or reconciliations. What we have demonstrated is a model of a family physician working WITH a pharmacist and iteratively have developed the balance that fits our context. It will be up to each pair or group, even in our one clinic to develop their own comfort level between two equal professionals sharing responsibility for the same group of patients over time.

## 5. Conclusions

It is possible to establish a family physician–pharmacist collaboration that involves both providing direct patient care independently and in shared consultation. The pharmacist had at least one encounter with 159 patients between April 2014 and March 2020. During that time, the pharmacist completed 583 visits with 220 independent visits. There was a total of 1361 prescriptions written by the pharmacist.

## Figures and Tables

**Table 1:** Differences between family physicians and primary care physician [34].

Family Physician	Primary Care Physician
1. Continuous relationship with the patients	1. Practice is limited to selected medical condition
2.First contact physician for patients with any medical problems	2. Refer patients to other specialists who have medical condition outside of their scope of practice.
3. The family is a unit of care	3. Fragmented patient care
4. Coordinating with other specialists	4. Do not take the holistic approach
5. Screening and prevent diseases such as cancer	5. Greater risk of polypharmacy and drug interactions
6. Continuity of care	
7. Established long-term patient/physician relationship with the patient and family	
8. Use holistic approach	
9. Large volume of patients	
10. Diverse practice	

**Table 2:** Patient Characteristics.

Demographics	
Patients	159
Age range	19–94
Average age	62
Male	76
Female	83
**Past Medical History**	
HTN	57
Mental Health	32
DM	22
Chronic pain	22
Hypothyroidism	17
Dyslipidemia	14

**Table 3:** Number of visits and prescriptions.

Visit	2014	2015	2016	2017	2018	2019	2020	Total
Independent (%)	41(61)	21(21)	15(25)	20(25)	31 (30)	74(51)	18(69)	220(38)
Total visits	67	101	61	80	105	143	26	583
Prescriptions	97	121	162	207	282	353	139	1361
Prescription/visit	1.4	1.2	2.7	2.6	2.7	2.5	5.3	2.3

## Data Availability

Data is contained within the article.
